# Morphometric Analysis of Neocortical and Infratentorial Structures: Genetic and Environmental Insights from a Twin Neuroanatomical Study

**DOI:** 10.3390/medicina61020261

**Published:** 2025-02-04

**Authors:** Amirreza Alijanpourotaghsara, Arsalan Vessal, Amirmasoud Alijanpour, David Strelnikov, Marton Piroska, Aliz Persely, Zsofia Jokkel, Laszlo Szalontai, Bianka Forgo, Lajos Rudolf Kozak, Adam Bekesy-Szabo, Pal Maurovich-Horvat, David Laszlo Tarnoki, Adam Domonkos Tarnoki

**Affiliations:** 1 Medical Imaging Centre, Semmelweis University, 1082 Budapest, Hungary; amirrezaalijanpour1373@gmail.com (A.A.); vessalarsalan@gmail.com (A.V.); amirmasoudalijanpour@gmail.com (A.A.); david.strelnikov17@gmail.com (D.S.); piroskamarton94@gmail.com (M.P.); aliz.persely@gmail.com (A.P.); zsofijokkel@gmail.com (Z.J.); laszlo.szalontai.med@gmail.com (L.S.); lkozak@mrkk.sote.hu (L.R.K.); szabadam@gmail.com (A.B.-S.); maurovich-horvat.pal@semmelweis.hu (P.M.-H.); tarnoki4@gmail.com (D.L.T.); 2 Department of Clinical Neuroscience, Karolinska Institutet, SE-171 77 Stockholm, Sweden; fbia021@gmail.com; 3 Department of Neuroradiology, Karolinska University Hospital, SE-171 77 Stockholm, Sweden; 4 Faculty of Medicine and Health, School of Medical Sciences, Örebro University, SE-701 82 Örebro, Sweden

**Keywords:** brain morphology, genetic factors, cortical thickness, heritability, neuroimaging

## Abstract

*Background and Objective:* Brain morphometry is shaped by a complex interplay of genetic and environmental factors, including physiological and neuropsychiatric conditions. These influences can vary across distinct brain regions, yet the precise contributions of genetics and environment to regional variation in healthy brains remain poorly understood. This study examines the heritability of specific brain structures to provide deeper insights into their development. *Materials and Methods:* We studied 118 healthy adult twins from the Hungarian Twin Registry using T1-weighted magnetic resonance imaging (T1W MRI) and the volBrain pipeline for structural measurements. *Results:* In all regions, monozygotic (MZ) twins showed a higher resemblance than dizygotic (DZ) twins in total brainstem and cerebellar volumes, with significant heritability (A: 90.5–92.6%) and minimal unique environmental effects (E: <1%). For supratentorial regions, regarding the total gray matter volume, all regions exhibited high heritability (A: 74.5–92.4%) and minimal environmental influence (E: <1.5%). In average cortical thickness analysis, the frontal lobe, temporal lobe, and pre-central gyrus were influenced by shared and unique environmental factors (C: 63–66.5%; E: 33.4–37%), whereas genetics were more prominent in the parietal lobe, occipital lobe, and post-central gyrus (A: 67.7–85%; E: 15–32.3%). *Conclusions:* Genetics strongly influence cortical gray matter volume in supratentorial regions (both total and regional), as well as the total brainstem volume and the total and cortical gray matter volumes of the cerebellum in infratentorial regions. This genetic influence extends to the average cortical thickness of the parietal lobe, post-central gyrus, and occipital lobe, while the frontal lobe, temporal lobe, and pre-central gyrus are more affected by environmental factors. These findings emphasize the importance of understanding region-specific genetic and environmental contributions to brain structure, which could guide personalized therapeutic and preventive strategies for neurological conditions.

## 1. Introduction

The human brain has been a focal point of neuroscientific investigation for decades, with significant emphasis on understanding the dynamic interplay between brain structure and neuropsychiatric conditions. Among neocortical regions, noteworthy alterations in cortical gray matter volume and thickness have been observed as a result of aging and various disorders. Multiple studies have highlighted the profound effects of conditions such as attention-deficit/hyperactivity disorder (ADHD) and normative patterns of cortical development. Furthermore, changes in cortical structure have been associated with a range of conditions, including bipolar disorder and obsessive–compulsive disorder (OCD) [1,2,3,4,5,6].

Within the cerebral cortex, the pre-central and post-central gyri emerge as pivotal regions demonstrating significant structural changes in numerous neuropsychiatric disorders. Studies have consistently illuminated morphological shifts in these regions, linking them to conditions such as bipolar disorder, amputations, and memory impairment [7,8]. These gyri are integral to understanding motor and sensory processing and their implications in disorders like OCD and glaucoma [9,10,11,12]. Moving posteriorly, the cerebellum and brainstem stand out as regions undergoing significant morphological changes due to aging and neuropsychiatric syndromes; these volume shifts are especially observed in Parkinsonian syndromes and fragile X premutation [13,14].

Twin studies offer an exceptional framework for differentiating the roles of genetics and environment in shaping human traits. Monozygotic (MZ) twins, or identical twins, originate from a single fertilized egg that splits into two during early development, sharing nearly identical genetic information. In contrast, dizygotic (DZ) twins arise from two separate fertilized eggs, inheriting approximately 50% of the same genetic material. Both twin types typically experience similar environmental influences, though individual variances persist. This unique setup allows researchers to explore the relative contributions of nature and nurture to various phenotypes [15].

Neuroanatomical studies in twins provide valuable insights into the genetic and environmental effects on brain structure and their potential links to neuropsychiatric disorders. For instance, Hardan et al. identified a smaller caudate volume in an MZ twin with autism compared to his healthy sibling, suggesting neuroanatomical correlates of autism spectrum disorder [16]. This finding was validated by Mitchell et al., who also reported structural brain differences in MZ twins discordant for autism, highlighting the role of neuroanatomic alterations in the disorder [17]. Additionally, Westeinde et al. examined neuroanatomical correlates of restricted and repetitive behaviors in twins, underscoring the differential involvement of specific brain regions in neurodevelopmental disorders [18].

In this study, we assess the magnitude of genetic and environmental influences on neocortical and infratentorial structures, motivated by their distinct functional roles and differing developmental trajectories. Neocortical regions, including the brain’s major lobes and sensorimotor cortex, are crucial for higher-order cognitive functions, sensory processing, and motor control. Infratentorial structures, comprising the brainstem, cerebellum, and cerebellar cortex, are vital for motor coordination, autonomic regulation, and various neuropsychiatric processes. Our previous research on white matter hyperintensities (WMHs) revealed divergent heritability patterns between supratentorial and infratentorial regions, underscoring the need to investigate the independent influences of genetic and environmental factors on these distinct anatomical structures [19].

Using a healthy adult twin cohort, we analyze cortical gray matter volume and average cortical thickness in the neocortex, alongside volumetric measures of the brainstem, cerebellum, and cerebellar cortex. This approach isolates the effects of age and environmental factors on the healthy brain, excluding the confounding influence of underlying pathology. Employing 3-Tesla MRI and an automated volumetry pipeline, this study aims to provide a nuanced and precise understanding of healthy brain morphology, exceeding the scope of many previous investigations.

## 2. Materials and Methods

### 2.1. Participants and Demographics

We investigated 118 asymptomatic Caucasian adult twins (56 pairs and 2 triplets) sourced from the Hungarian Twin Registry [20]. None of the participants had a history of cerebrovascular or neurodegenerative diseases. For analysis, the triplets were treated as three separate twin pairs. This approach yielded 62 twin pairs: 43 monozygotic (MZ) and 19 dizygotic (DZ). We excluded two twin pairs due to missed appointments, another for subpar imaging quality, and one pair for being opposite-sex DZ twins. The participants’ median age was 50 ± 27 years, with a 71.2% to 28.8% female-to-male ratio. The study received approval from the local Ethics Committee (Semmelweis University, TUKEB 189-1/2014; amendments: 10 October 2016, 7 December 2018). All participants provided informed consent, adhering to the Declaration of Helsinki. Zygosity was ascertained using a seven-item self-reported questionnaire [21]. Data on history and risk factors were collected via a questionnaire, covering aspects such as height, body weight, BMI, smoking status, hypertension, hyperlipidemia, and diabetes. The smoking category encompassed both current and former smokers. Participants underwent MRI examinations, provided there were no contraindications. Individuals were excluded if they had recent immunosuppressive or immunomodulant therapy, had undergone chemotherapy within the past year, had major surgeries or blood transfusions in the last two months, were pregnant, or were breastfeeding. Similarly, those with pacemakers, Implantable Cardioverter Defibrillators (ICDs), other implanted devices, magnetic metal foreign bodies, or a history of claustrophobia were not included.

### 2.2. MRI Acquisition

Three-dimensional (3D) T1-weighted images were acquired for all participants without contrast agents. The scans were conducted at the Semmelweis University MR Research Centre using a Philips Ingenia 3T scanner (Philips Healthcare, Best, The Netherlands). The Philips scanner was set with the following parameters: TE/TR of 140/9000 ms, a flip angle of 88°, a 290 × 336 × 336 matrix, an in-plane resolution of 0.8333 × 0.8333, and a slice thickness of 0.6 mm.

### 2.3. Image Processing

The absolute volumes and average thickness of cortical structures—including the cerebrum, cerebellum, frontal, temporal, parietal, and occipital lobes, as well as the pre-central and post-central gyri—were quantified. The 3D T1-weighted images were converted from DICOM (Digital Imaging and Communications in Medicine) to NIfTI (Neuroimaging Informatics Technology Initiative) format using the DCM2NII converter (http://www.mricro.com, mricron; Chris Rorden, Columbia, SC, USA, accessed on May 2023 and used throughout the analysis period). All subsequent image processing was conducted in this format [22].

#### Volumetric Analysis with volBrain

**Figure 1:**
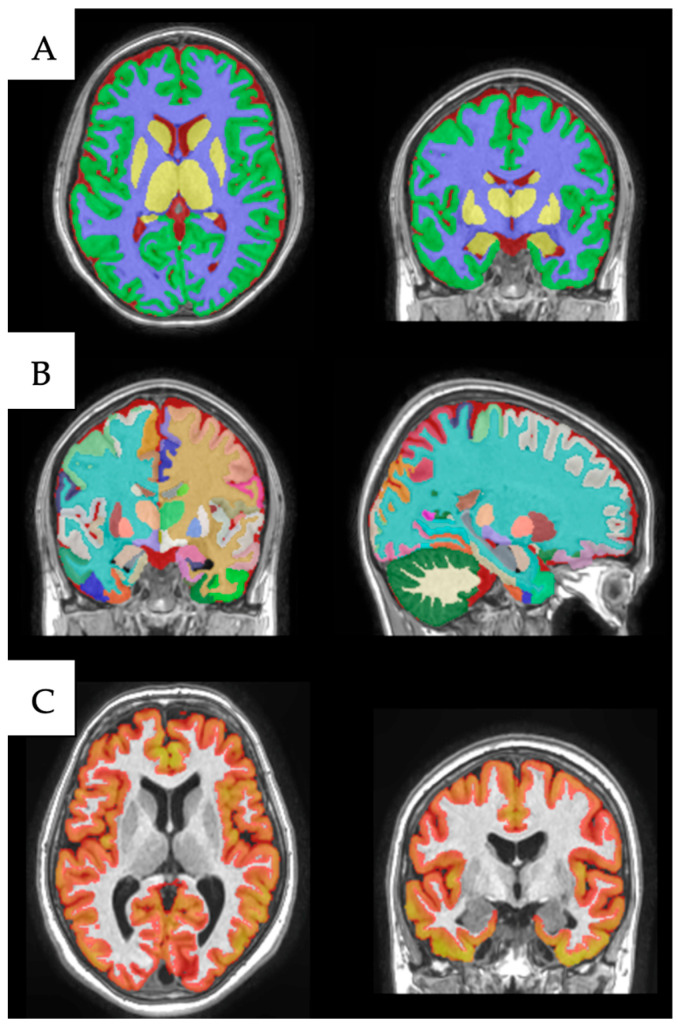
The multi-step parcellation, classification, and segmentation of brain tissue using the volBrain pipeline. (**A**) Tissue segmentation into white matter, cortical and subcortical gray matter, and brainstem. (**B**) Structure segmentation for differentiating cortical and subcortical structures into various nuclei and subsections. (**C**) Cortical gray matter differentiation and segmentation.

The volBrain platform (https://www.volbrain.upv.es, accessed on May 2023 and utilized over the following months) was utilized to determine the volume of subcortical brain regions. volBrain is an autonomous MRI brain volumetry system that operates without manual intervention to execute tasks and produce reports [23,24]. The system first de-noises the scans, then applies an inhomogeneity correction, registers to the Montreal Neurological Institute (MNI) space, conducts intensity normalization, and extracts the intracranial cavity. Subsequently, tissue segmentation is executed using a multi-template fusion atlas approach derived from the manual segmentation of 50 subjects [25]. The volume of pertinent structures is then gauged in cubic centimeters (cm^3^), considering age and gender for normative developmental values. Figure 1 illustrates the comprehensive outcome of the volBrain analysis based on the subject’s T1-weighted images.

**Figure 2:**
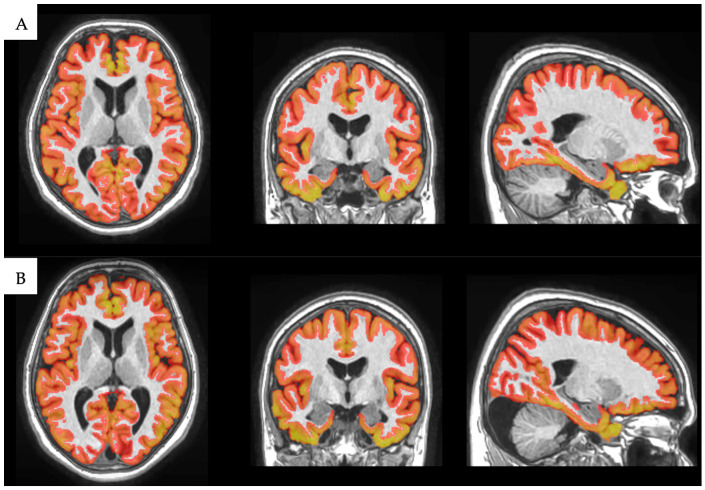
Cortical segmentation of 73-year-old female MZ twins reveals visible similarities in their cortical structures. Based on our analysis, the total cortical gray matter volume for twin (**A**) was 447,015.2 cm^3^, and for twin (**B**), it was 445,128.5 cm^3^. Image courtesy of the Semmelweis University Medical Imaging Centre.

**Figure 3:**
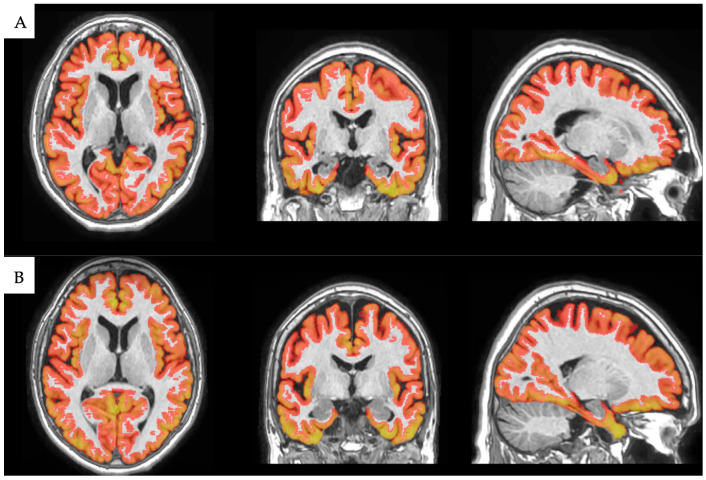
Cortical segmentation of 74-year-old female DZ twins displays distinct differences in several cortical regions. Based on our study, the total cortical gray matter volume for twin (**A**) was 481,961.4 cm^3^, and for twin (**B**), it was 413,194.5 cm^3^. These disparities are also evident in sub-regions; for instance, the total parietal lobe volume for twin (**A**) stood at 91,415.4 cm^3^, while for twin (**B**), it was 75,010.6 cm^3^. Image courtesy of the Semmelweis University Medical Imaging Centre.

In this study, as we focused on the cortical gray matter volume and average thickness of the major brain regions, we utilized the results of the cortical thickness analysis. Figure 2 and Figure 3 showcase examples of cortical segmentation derived from high-resolution T1-weighted MRI images in our study for a monozygotic (MZ) and a dizygotic (DZ) twin pair.

### 2.4. Statistical Analysis

#### 2.4.1. Descriptive Statistics

Continuous variables underwent normality testing using the Shapiro–Wilk test. For normally distributed variables, such as BMI, the means were compared between MZ and DZ twins using an independent-sample *t*-test and presented as mean ± standard deviation (SD). Non-normally distributed variables, like participant age, were compared using the Mann–Whitney U-test and depicted as median ± interquartile range (IQR). Categorical variables, including sex, diabetes, smoking, hypertension, and hyperlipidemia, were assessed with the chi-squared test and represented as frequencies and percentages. Additionally, the absolute volumes of subcortical structures were compared between MZ and DZ twins. A *p*-value below 0.05 was deemed significant. All analyses were performed using SPSS software (IBM Corp., IBM SPSS Statistics, Version 28.0. Armonk, NY, USA).

#### 2.4.2. Twin Modeling of Genetic and Environmental Influences

The variance in phenotype among individuals is attributed to genetic and environmental effects. Genetic influences are divided into additive genetic factors (A), which cover polygenetic inheritance, and dominant genetic factors (D), like epistasis. On the environmental side, influences can be common (C) or unique (E). Common environmental factors refer to experiences shared by twins, such as a similar childhood diet, exposure to parental smoking, air pollution, and sharing a womb. Non-shared or unique environmental factors relate to individual experiences distinct to each twin and not shared by their sibling, such as individual smoking habits, different physical activities, specific occupational exposures, and separate illnesses [26].

Classical twin studies operate on the premise that MZ twins share approximately 100% of their genes, while DZ twins share around 50%. Additionally, both MZ and DZ twins share all their common environmental influences but none of their unique ones. Heritability estimates were derived by comparing intra-pair correlations between MZ and DZ twins. A higher intra-pair correlation in MZ twins than in DZ twins suggests a genetic component. Conversely, the variance was ascribed to environmental influences if the correlations were similar for both MZ and DZ twins [27]. Univariate quantitative genetic modeling was employed to decompose the variance into additive genetic (A), common environmental (C), and unique environmental components (E), with adjustments for age and sex. Due to the confounding effects, dominant genetic factors (D) and common environmental factors (C) could not be estimated simultaneously in the model. Thus, the best-fitting ACE model was selected.

To determine the most appropriate model (ACE, AE, or CE) for each trait, we employed a data-driven approach using the likelihood-ratio test and variance component estimates. Traits with negligible common environmental variance (C) were best fit by the AE model, while traits with minimal genetic variance (A) were fit using the CE model. The ACE model was retained for traits where both genetic and shared environmental contributions were significant. This approach ensures parsimony while accurately capturing the variance components for each trait.

A likelihood-ratio test was used to compare the different models. *p*-values greater than 0.05 indicated that there was no significant difference between the base and the reduced models, justifying the use of the more parsimonious AE or CE model.

## 3. Results

### 3.1. Descriptive Findings

**Figure 4:**
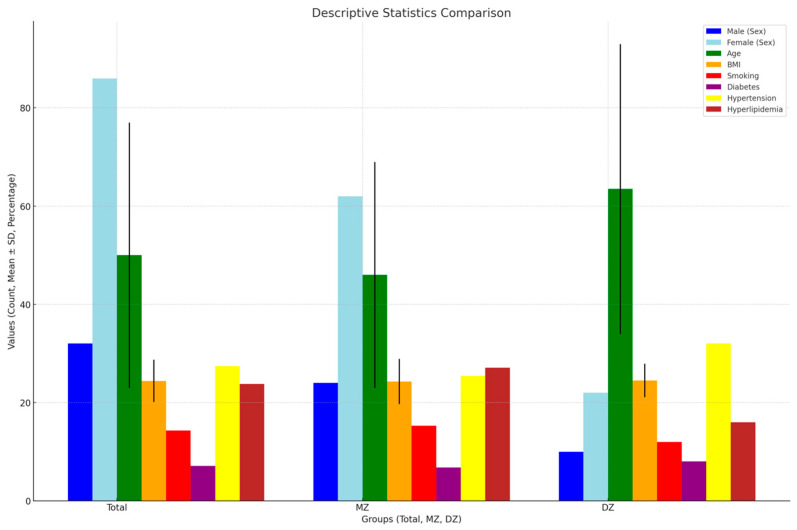
Descriptive statistics comparison: The figure presents the distribution of various descriptive statistics for the total sample, as well as MZ and DZ twins. Variables include sex distribution, age (mean ± SD), BMI (mean ± SD), and the prevalence of smoking, diabetes, hypertension, and hyperlipidemia. The X-axis represents the groups (total, MZ, DZ), and the Y-axis represents values (count, mean ± SD, percentage).

This study included 118 participants, divided into 86 monozygotic (MZ) and 32 dizygotic (DZ) twins. The gender distribution comprised 32 males and 86 females, with 24 males and 62 females in the MZ group and 10 males and 22 females in the DZ group. The median age for the MZ twins was 46 years, and for the DZ twins, it was 64 years, showing a statistically significant difference (*p*-value = 0.03). Despite this, no significant differences were found in Body Mass Index (BMI), smoking habits, diabetes, hypertension, or hyperlipidemia between the groups. Figure 4 provides a visual summary of the descriptive statistics. Supplementary Table S1 offers a more detailed breakdown of the variables, including additional metrics not captured in the figure.

#### 3.1.1. Intra-Pair Correlation and Univariate Model Analysis of Cortical Gray Matter Volume

**Figure 5:**
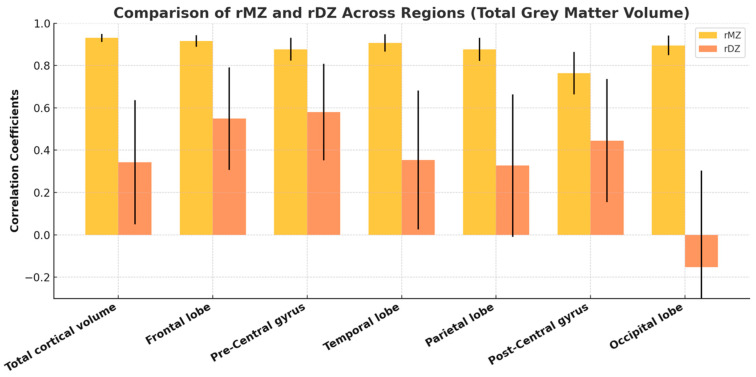
A grouped bar chart with error bars comparing the correlation coefficients (rMZ and rDZ) for total gray matter volume across various brain regions. The bars represent the mean correlation coefficients, while the error bars indicate the standard error of the mean (SEM) or 95% confidence intervals, reflecting variability within the data. The chart highlights differences in heritability patterns across cortical regions.

**Figure 6:**
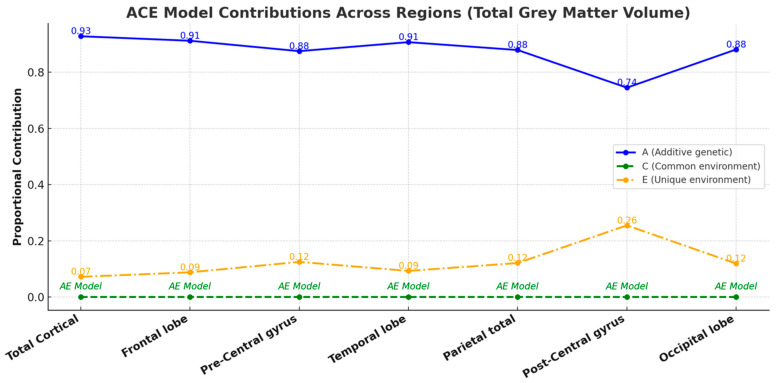
A line chart illustrating the proportional contributions of additive genetic (A), common environmental (C), and unique environmental (E) factors to the total gray matter volume across brain regions, as determined by the ACE model. Each line represents the contribution of a specific factor (A, C, or E) to the variance, with numerical annotations indicating precise proportional values. Regions with negligible common environmental effects (C) default to the AE model, underscoring the predominance of genetic and unique environmental influences. This visual representation emphasizes the significant genetic and environmental contributions to the variability of cortical gray matter volume across different regions.

The correlation coefficients for total and regional cortical gray matter volume consistently showed higher values in monozygotic (MZ) twins compared to dizygotic (DZ) twins across all categories (Figure 5), suggesting a significant genetic influence on cortical gray matter volume across these regions. The age- and sex-adjusted univariate analysis showed substantial heritability (A) for total brain and all regional volumes. Due to the negligible contributions from common environmental factors (C), the more fitted AE model was used for all structures. The fit of this model showed no significant difference compared to the general ACE model. Unique environmental variance was a minor contributor across all variables (Figure 6). The detailed statistical data, including confidence intervals, are provided in Supplementary Materials Table S2.

#### 3.1.2. Intra-Pair Correlation and Univariate Model Analysis of Key Average Cortical Thickness Measurements

**Figure 7:**
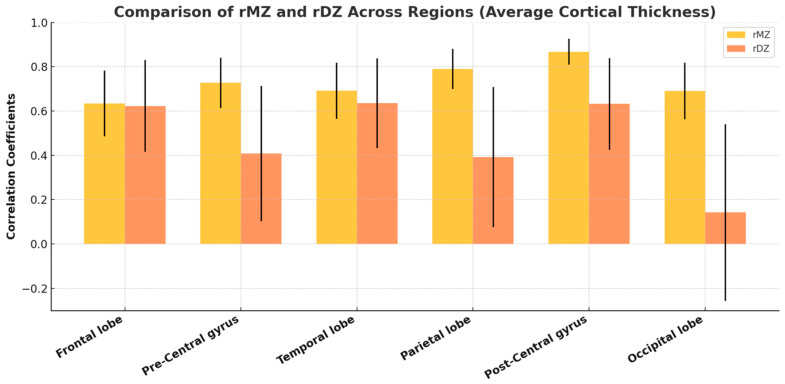
A grouped bar chart with error bars comparing the correlation coefficients (rMZ and rDZ) for average cortical thickness across various brain regions. The bars represent the mean correlation coefficients, while the error bars indicate the standard error of the mean (SEM) or 95% confidence intervals, reflecting variability within the data.

**Figure 8:**
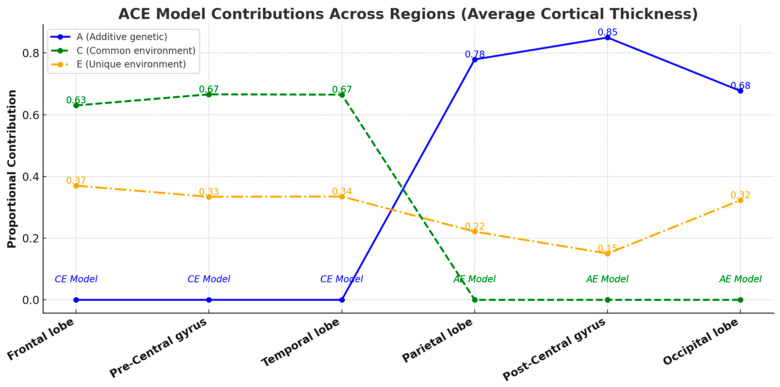
A line chart illustrating the proportional contributions of A, C, and E factors to the average cortical thickness across brain regions, as determined by the ACE model. Each line represents the contribution of a specific factor to the variance, with numerical annotations indicating precise proportional values. Regions with negligible additive genetic effects (A) or common environmental effects (C) default to the CE or AE models, respectively, highlighting the variability in factor dominance across regions. This visual representation underscores the significant roles of genetic and environmental influences in determining cortical thickness variability across different brain regions.

Intra-pair correlation coefficients for key cortical thickness measurements consistently showed higher values in MZ twins compared to DZ twins across all categories (Figure 7), suggesting a genetic influence on cortical thickness, although the relative contributions of genetic and environmental factors varied across regions. The age- and sex-adjusted univariate analysis revealed significant A for the average thickness of the parietal lobe, post-central gyrus, and occipital lobe. Due to the negligible contributions from C, the AE model was preferred for these areas. This model’s fit was comparable to the broader ACE model. For the frontal lobe, temporal lobe, and pre-central gyrus, the CE model was more appropriate, indicating significant C contributions with values of 0.63, 0.665, and 0.666, respectively. E played a minor role across all evaluated regions. A detailed account of the heritability analysis for these cortical thickness measurements is provided in Figure 8. The detailed statistical data, including confidence intervals, are provided in Supplementary Materials Table S3.

#### 3.1.3. Intra-Pair Correlation and Univariate Model Analysis of Infratentorial Structure Measurements

**Figure 9:**
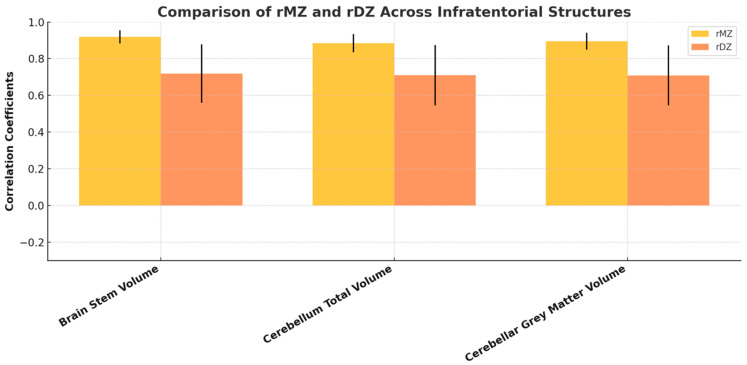
A grouped bar chart with error bars illustrating the correlation coefficients (rMZ and rDZ) for infratentorial structures. Each bar represents the mean correlation coefficient, while the error bars denote the standard error of the mean (SEM) or 95% confidence intervals, highlighting the variability within the data.

**Figure 10:**
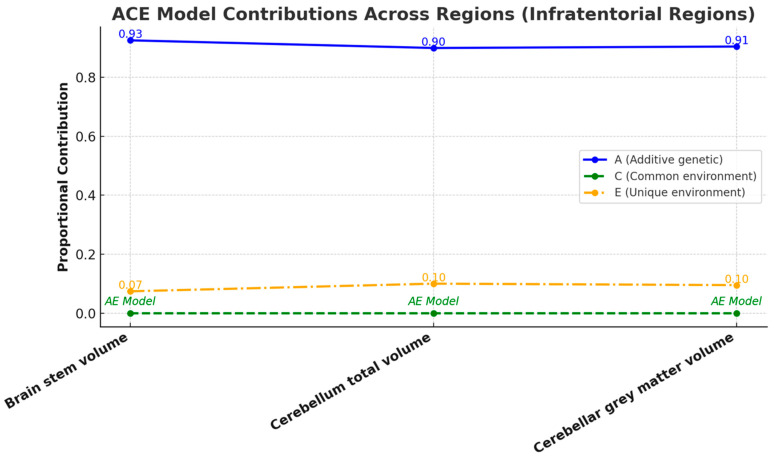
A line chart illustrating the proportional contributions of A, C, and E factors to the volume of infratentorial structures, as determined by the ACE model. Each line represents the contribution of a specific factor to the variance, with numerical annotations indicating precise proportional values. Regions with negligible C default to the AE model, highlighting the predominance of genetic and unique environmental influences. This visual representation emphasizes the significant genetic and environmental contributions to the variability of infratentorial structure volumes.

The infratentorial structure volumes consistently demonstrated higher correlation coefficients in MZ twins compared to DZ twins across all categories (Figure 9), suggesting a significant genetic influence on the volume of these structures. Age- and sex-adjusted univariate analysis revealed significant heritability (A) for all infratentorial regional volumes. Given the minimal contributions from common environmental factors (C), the AE model was deemed more appropriate for all structures. The AE model fit showed no significant deviation from the general ACE model. Unique environmental variance (E) contributed minimally to all variables (Figure 10). Detailed statistical results, including confidence intervals, are available in Supplementary Materials Table S4.

This analysis underscores distinct patterns of genetic and environmental influences between supratentorial total and regional cortical gray matter volume and average thickness as well as infratentorial regions. These findings highlight the differences in heritability across these anatomical areas, reflecting the unique genetic and environmental contributions to their structural variability.

## 4. Discussion

In this neuroanatomical investigation, we assessed the heritability of infratentorial structures, including brainstem total volume and cerebellar total and cortical volume, as well as neocortical regions with a detailed examination of specific subsections, such as the pre- and post-central gyri. Our analyses utilized 3T T1-weighted MRI imaging combined with volBrain, a fully automated and efficient segmentation method for precise and rapid analysis.

A key strength of this study lies in its comparative analysis of supratentorial and infratentorial structures, which provides novel insights into the unique heritability patterns of these regions. By evaluating these areas together, we captured the region-specific contributions of genetic and environmental factors to brain morphology. Specifically, our analysis focused on three distinct components: cortical gray matter volume for total and major anatomical regions, the average cortical gray matter thickness across these regions, and the volumetric properties of infratentorial regions, including brainstem volume, cerebellar total volume, and cortical gray matter volume.

We found that genetic factors exerted a strong influence on total and regional cortical gray matter volumes across regions, with A-values consistently high (i.e., 0.912 for the frontal lobe, 0.907 for the temporal lobe, and 0.879 for the parietal lobe) (Table S2). These findings underscore the predominant role of heritability in shaping brain morphology. However, for average cortical thickness, we observed a more nuanced pattern, with genetic contributions remaining strong in regions such as the parietal lobe (A = 0.879) and occipital lobe (A = 0.881), while shared environmental factors played a more substantial role in the frontal and temporal lobes.

Additionally, focusing on healthy adult twins free of pathological conditions eliminates confounding factors and ensures that observed variations are not influenced by disease-related morphological changes, thereby providing a clearer understanding of the genetic and environmental contributions to brain structure and establishing a solid framework for understanding baseline heritability patterns. While prior studies have largely emphasized subcortical nuclei, our research shifts the focus to cortical regions while incorporating infratentorial structures for a more integrated perspective. This dual approach allows us to address gaps in the literature by emphasizing the genetic dominance in certain brain regions while uncovering areas where environmental factors are more influential.

The reliability of our findings is further supported by volBrain’s precision, comparable to CAT12, a gold standard for brain segmentation [28].

### 4.1. Comparison with Prior WMH Heritability Findings

In our prior study, we found a notable genetic influence on the presence of WMH in the infratentorial regions (brainstem and cerebellum), while environmental factors were more prominent in the occurrence of WMH in supratentorial regions [19]. This finding is relevant to our current study as it underscores the genetic determinants of brain structures within the infratentorial region, complementing our observation of strong genetic influences on the volume of infratentorial structures. Conversely, in supratentorial regions, we observed a hybrid effect. Although the effect of genetics was more prominent for the cortical gray matter volume of subregions as well as the average cortical thickness of the parietal lobe, occipital lobe, and post-central gyrus, environmental factors had a more significant impact on the cortical thickness of areas such as the frontal and temporal lobes. This pattern aligns with the WMH heritability findings, where environmental influences are more pronounced in these regions.

### 4.2. Clinical Implications of Heritability Findings

The heritability of cortical brain structures identified in our study provides valuable insights for clinical applications. By pinpointing regions with pronounced genetic determinants, we pave the way for earlier detection of susceptibility to neurological conditions, especially in those with familial predispositions [29,30]. This knowledge can lead to tailored therapeutic interventions, optimizing patient outcomes by leveraging the interplay of genetics and environment. The potential for guiding pharmaceutical research targeting genetically influenced brain regions is also vast [31]. Lastly, for neuropsychiatric disorders marked by structural brain differences [2,32], our findings offer a deeper understanding of their origins and possible treatment avenues, bridging genetics, neuroanatomy, and clinical practice for future patient-centric endeavors.

### 4.3. Environmental Dominance in Temporal and Frontal Regions

A study on lifelong bilingualism revealed that immersive language training resulted in increased thickness in the frontal lobe and superior temporal gyrus, as well as an enlarged hippocampal volume, underscoring the potential of language experiences as influential common environmental factors shaping the structural characteristics of the frontal lobe [33].

Numerous studies have highlighted the various factors influencing brain morphometry, particularly in the temporal lobe. For instance, a study on cerebral correlates of cognitive aging highlighted gray–white matter differentiation specifically in the medial temporal lobes [34]. Another investigation associated environmental risks and polygenic loading for schizophrenia with pronounced decreases in gray matter volumes, with a noteworthy emphasis on the temporal lobe [35].

Additional studies have emphasized the impact of environmental factors on gray matter volume across several brain regions. Smoking and alcohol dependence have been correlated with diminished gray matter volumes and densities, particularly in the temporal lobes, prefrontal cortex, anterior cingulate, parietal cortex, and cerebellum [36]. Similarly, a study on obstructive sleep apnea pointed to reductions in gray matter volume or thickness prominently in the temporal lobe, along with the prefrontal cortex and subcortical structures such as the hippocampus, thalamus, and cerebellum [37].

Individuals with developmental prosopagnosia exhibited diminished gray matter volume specifically in the right anterior inferior temporal lobe and superior temporal sulcus/middle temporal gyrus [38]. Furthermore, Han et al. (2015) associated lower gray matter volume in the medial temporal lobe and frontal lobe regions with an increased susceptibility to scams among older adults [39]. The above-mentioned studies further support the findings of this analysis, demonstrating that environmental factors exert a more pronounced effect on the temporal and frontal regions of the brain as described in Tables S2 and S3.

### 4.4. Genetic Influence in the Parietal Region

Numerous studies have delved into factors influencing gray matter volume and cortical thickness, with a recurrent emphasis on the parietal lobe and post-central gyrus. As shown in Table S2, the parietal lobe exhibited high heritability (A = 0.879), supporting the strong genetic influence on structural variation in this region. One study found that Parkinson’s disease (PD) patients with visual hallucinations (VHs) exhibited reductions in gray matter volume, specifically in the superior parietal lobe, compared to controls and non-hallucinating PD patients [40]. This suggests a possible structural basis in these regions for VH in PD patients. In a related vein, patients with longstanding type 1 diabetes mellitus showed reduced cortical thickness prominently in the post-central and superior parietal gyrus [41]. Further emphasizing the significance of the parietal lobe, another study identified mother–child similarities in cortical thickness measures of the inferior parietal lobe, pointing to the presence of late-acting genetic factors that promote local cortical thinning [42]. Another study highlighted that genetic factors significantly influence gray matter thickness and volume, especially in parietal regions [43]. Outside these regions, it is also worth noting findings from another research, which associated cardiovascular disease risk factors (CVD-RFs) with variations in gray matter measures, illustrating the complex interplay between genetics and environmental exposures in shaping brain morphology [44].

### 4.5. Genetic Influence in the Occipital Region

Table S2 demonstrates the significant genetic contributions to brain morphology, with high A-values observed across cortical and infratentorial regions. For instance, genetic influence was particularly strong in the occipital lobe (A = 0.881) and parietal lobe (A = 0.879), highlighting their heritable nature. This finding is consistent with previous studies showing high heritability estimates for occipital lobe total volume (67%) and parietal regions associated with visuospatial processing. This research has shown heritability estimates for gray matter volume in various brain regions.

A study was conducted on 236 older twins with a focus on the heritability of 53 brain global and lobar volumetric measures [45]. The result of the brain MRI analysis and volumetric measures showed a significant influence of genetic factors on brain structure in older adults. The heritability estimates were 87% for occipital lobe white matter volume and 67% for occipital lobe total volume.

Another study explored the genetic and environmental impacts on gray matter volume in twin children and adolescents [46]. In this study, the gray matter volumes of 58 pairs of twins between 12 and 18 years of age were measured by MRI. The results of this study show that the gray matter volume was mostly affected by genetics in the frontal, parietal, occipital, and lateral temporal lobes. The effect of the heritability factor on the occipital lobe gray matter volume was close to 70% (A: 0.7).

One study focusing on the older adult twin population also exhibited a high to moderate level of genetic influence on the temporal, parietal, frontal, and occipital lobes [47].

According to our study, the genetic factor’s influence on the cortical thickness of the occipital lobe was prominent. Previous studies found a similar pattern of influence.

A twin study conducted by Gilmore et al. revealed that heritability estimates for cortical thickness in different brain regions, including the occipital lobe, increased from late childhood through adolescence, suggesting that genetic influences become increasingly significant as the brain matures [48]. This is consistent with findings from Yang et al., who reported that genetic factors markedly affect cortical thickness in individuals with schizophrenia, particularly in regions associated with visual processing, including the occipital lobe [49].

### 4.6. Genetic Dominance on the Volume of Infratentorial Structures

Lastly, our study observed a strong genetic influence on the volume of the cerebellum and brainstem. This observation aligns with findings from various neurological contexts. In spinocerebellar ataxia type 2 (SCA2), degeneration of the cerebellum and brainstem, notably the pontine volumes and the anteroposterior diameter of the pons, has been documented [50].

Migraine patients without aura have exhibited smaller cerebellum and brainstem volumes compared to healthy counterparts [51].

Our study presents a few notable limitations. First, the twin sample size is relatively small, which may limit the statistical power and generalizability of our findings. Specifically, the smaller proportion of dizygotic twins compared to monozygotic twins may restrict the ability to conduct robust subgroup analyses and reduce generalizability when comparing across zygosity groups. While our results provide valuable insights into the heritability of brain structures, a larger sample size would allow for more robust estimates and the ability to detect subtler effects.

Second, the demographic composition of our sample, which consisted of healthy middle-aged twins, may constrain the applicability of our findings to other populations, such as younger or older individuals, individuals with neurological disorders, or those from more diverse socioeconomic or ethnic backgrounds. Future studies using broader and more diverse cohorts could provide a more comprehensive understanding of genetic and environmental contributions to brain morphology across different populations.

Third, regarding the use of the volBrain program, while its absence of a manual correction feature might introduce potential artifacts, it is essential to highlight the significant advantages it offers as an automated segmentation tool. The benefits of using volBrain, in terms of efficiency and standardized processing, generally outweigh these concerns. To address potential issues, we manually reviewed the images to identify and correct any visible artifacts before proceeding with segmentation. It is well documented that automated platforms can face challenges when segmenting smaller, complex structures, which could introduce measurement errors. However, in our study, we predominantly focused on relatively larger brain segments and structures, which likely reduced the impact of such errors.

## 5. Conclusions

Our neuroanatomical study on healthy adult twins demonstrates a strong genetic influence on the cortical gray matter volume of supratentorial regions, both total and regional, as well as on infratentorial regions, including the total volume of the brainstem and the total and cortical gray matter volume of the cerebellum. This genetic influence also extends to the average cortical thickness of the parietal lobe, post-central gyrus, and occipital lobe. Conversely, the average cortical thickness of the temporal lobe, frontal lobe, and pre-central gyrus was more strongly influenced by common and unique environmental factors than genetic determinants.

Using ACE/AE/CE models, we identified significant genetic contributions (both additive and possibly dominant) to these measures. A similar genetic effect pattern was observed for infratentorial regions, while a hybrid genetic–environmental effect was evident in supratentorial regions.

These findings highlight the importance of further research to identify specific genes that influence regional brain volumes and cortical thickness, as well as shared environmental factors, such as in utero conditions, that impact cortical thickness in specific regions. Validation in larger twin cohorts is essential to strengthen these results. A better understanding of the genetic and environmental determinants of brain structure could inform targeted interventions for neurological conditions, including tailoring dementia treatments to account for the regional contributions of genetic and environmental factors.

## Data Availability

Data are available upon request.

## Supplementary Materials

The following supporting information can be downloaded at https://www.mdpi.com/article/10.3390/medicina61020261/s1. Table S1: Characteristics of the MZ and DZ twin study populations; Table S2: Intra-pair correlation coefficients and univariate analysis of total brain and regional volumes; Table S3: Analysis of cortical thickness measurements; Table S4: Analysis of infratentorial structure measurements.

## Abbreviations

The following abbreviations are used in this manuscript:

MZ	monozygotic
DZ	dizygotic
OCD	obsessive–compulsive disorder
ADHD	attention-deficit/hyperactivity disorder
MRI	magnetic resonance imaging
T1W	T1-weighted
WMH	white matter hyperintensity
BMI	Body Mass Index
ICD	Implantable Cardioverter Defibrillator
DICOM	Digital Imaging and Communications in Medicine
NIFTI	Neuroimaging Informatics Technology Initiative
SD	standard deviation
IQR	interquartile range
ACE	Additive, Common, and Unique Environmental model
rMZ	Correlation coefficient for monozygotic twins
rDZ	Correlation coefficient for dizygotic twins
MNI	Montreal Neurological Institute
