# Supplementary material for: Morphometric Analysis of Neocortical and Infratentorial Structures: Genetic and Environmental Insights from a Twin Neuroanatomical Study
Source: Medicina (Kaunas). 2025 Feb 4;61(2):261. doi: 10.3390/medicina61020261 (PMC11857582; doi:10.3390/medicina61020261)
Supplement: Supplementary file 1 [file medicina-61-00261-s001.zip › medicina-3431868-supplementary.pdf]

**Supplementary Material for " Morphometric Analysis of Neocortical and Infratentorial Structures: Genetic and Environmental Insights from a Twin Neuroanatomical Study"**

This supplementary material provides additional detailed statistical analyses and visualizations to support the main findings reported in the article "Morphometric Analysis of Neocortical and Infratentorial Structures: Genetic and Environmental Insights from a Twin Neuroanatomical Study." The tables below present descriptive statistics data, intra-pair correlation coefficients, and age- and sex-adjusted univariate analyses for total brain and regional volumes, as well as cortical gray matter volume and average thickness measurements across all regions in monozygotic (MZ) and dizygotic (DZ) twins.

- **Table S1: Characteristics of the MZ and DZ twin study populations.**

BMI: body mass index; DZ: dizygotic; MZ: monozygotic. Data are shown as mean ± standard deviation with continuous variables that had a normal distribution (BMI), and as median ± interquartile range with continuous variables that had a non-normal distribution (age). †: Results of the non-parametric Mann–Whitney U-test. All other p-values for continuous variables were calculated using the independent samples t-test. p-Values for dichotomous variables were calculated using the chi-squared test. p-Values less than 0.05 were significant. Significant results are marked with an asterisk *.

| Variable | Total (n = 118) | MZ (n = 86) | DZ (n = 32) | *p* -Value |
|---|---|---|---|---|
| Zygosity (*n* MZ:*n* DZ) | 86:32 | - | - | - |
| Sex (male:female) | 32:86 | 24:62 | 10:22 | 0.9 |
| Age (years) | 50 ± 27 | 46 ± 23 | 63.5 ± 29.5 | 0.04 †,* |
| Body mass index (kg/m$^2$) | 24.4 ± 4.3 | 24.3 ± 4.6 | 24.5 ± 3.4 | 0.86 |
| Smoking, *n* (%) | 12 (14.3) | 9 (15.3) | 3 (12.0) | 0.7 |
| Diabetes, *n* (%) | 6 (7.1) | 4 (6.8) | 2 (8.0) | 0.84 |
| Hypertension, *n* (%) | 23 (27.4) | 15 (25.4) | 8 (32.0) | 0.54 |
| Hyperlipidemia, *n* (%) | 20 (23.8) | 16 (27.1) | 4 (16.0) | 0.27 |

- **Table S2: Intra-Pair Correlation Coefficients and Age- and Sex-Adjusted Univariate Analysis of Twins' Total Brain and Regional Volumes.**

This table presents the combined intra-pair correlation coefficients of total brain and regional volumes for monozygotic (MZ) and dizygotic (DZ) twins alongside the age- and sex-adjusted univariate analysis. Results and their corresponding 95% confidence intervals (95% CI) are detailed. P-values below 0.05 are deemed significant. All the volumes are quantified in cubic centimeters ($cm^3$). The table employs several essential notations: A signifies heritability; C indicates the shared environmental variance component; E represents the unique environmental variance component; rMZ denotes the intra-pair correlation coefficient for monozygotic twins; and rDZ stands for the intra-pair correlation coefficient for dizygotic twins. The model fit, also provided in the table, represents the p-value derived from the chi-squared test. This test is based on a comparative examination of model log-likelihood concerning the foundational ACE model.

The **AE model** was selected for all traits presented in this table based on the statistical fit assessed via the chi-squared test (model p-values derived from log-likelihood comparisons). The selection reflects negligible shared environmental variance (C) observed in the data, indicating that additive genetic (A) and unique environmental factors (E) accounted for most of the variance. The model fit, also provided in the table, represents the p-value derived from the chi-squared test comparing the AE model with the foundational ACE model.

| Variable | rMZ | rDZ | A | C | E | Model fit P-value |
|---|---|---|---|---|---|---|
| **Brain total volume** | 0.923 ( 0.879, 0.949 ) | 0.412 ( -0.004, 0.674 ) | 0.919 (0.873, 0.946) | 0 | 0.081 (0.054, 0.127) | 1 |
| **Cortical grey matter volume** | 0.931 ( 0.891, 0.95) | 0.343 ( -0.092, 0.636 ) | 0.928 (0.886, 0.952) | 0 | 0.072 (0.048, 0.114) | 1 |
| **Cerebellar grey matter voluem** | 0.895 ( 0.816, 0.942) | 0.709 ( 0.402, 0.873) | 0.905 (0.84, 0.942) | 0 | 0.095 (0.058, 0.16) | 0.173 |
| **Brain stem volume** | 0.919 ( 0.857, 0.955 ) | 0.719 ( 0.418, 0.878 ) | 0.926 (0.875, 0.956) | 0 | 0.074 (0.044, 0.125) | 0.154 |
| **Cerebrum total volume** | 0.93 ( 0.89, 0.953 ) | 0.397 ( -0.023, 0.674 ) | 0.924 (0.88, 0.949) | 0 | 0.076 (0.051, 0.12) | 1 |
| **Cerebellum total volume** | 0.885 ( 0.797, 0.935 ) | 0.71 ( 0.402, 0.875 ) | 0.9 (0.833, 0.94) | 0 | 0.1 (0.06, 0.167) | 0.195 |
| **Frontal lobe total grey matter volume** | 0.916 ( 0.851, 0.944 ) | 0.549 ( 0.152, 0.792 ) | 0.912 (0.852, 0.947) | 0 | 0.088 (0.053, 0.148) | 0.644 |
| **Pre-central gyrus total grey matter volume** | 0.877 ( 0.784, 0.931 ) | 0.58 ( 0.199, 0.808 ) | 0.875 (0.791, 0.925) | 0 | 0.125 (0.075, 0.209) | 0.435 |
| **Temporal lobe total grey matter volume** | 0.907 ( 0.836, 0.948 ) | 0.354 ( -0.09, 0.682 ) | 0.907 (0.841, 0.945) | 0 | 0.093 (0.055, 0.159) | 1 |
| **Parietal total grey matter volume** | 0.876 ( 0.783, 0.931 ) | 0.327 ( -0.121, 0.664 ) | 0.879 (0.793, 0.928) | 0 | 0.121 (0.072, 0.207) | 1 |
| **Post-central gyrus total grey matter volume** | 0.764 ( 0.604, 0.864 ) | 0.445 ( 0.017, 0.736 ) | 0.745 (0.595, 0.841) | 0 | 0.255 (0.159, 0.405) | 0.528 |
| **Occipital lobe total grey matter volume** | 0.895 ( 0.815 0.941 ) | -0.153 ( -0.551 0.303 ) | 0.881 (0.795, 0.93) | 0 | 0.119 (0.07, 0.205) | 1 |

**Table S3: Intra-Pair Correlation Coefficients and Age- and Sex-Adjusted Univariate Analysis of Key Cortical Thickness Measurements in Twins.**

The table merges data from intra-pair correlation coefficients for cortical thickness measurements of key brain regions in monozygotic (MZ) and dizygotic (DZ) twins and the age- and sex-adjusted univariate analysis using volBrain. Results for both segments and their respective 95% confidence intervals (95% CI) are provided. Thickness measurements are denoted in millimeters (mm). For clarity, rMZ signifies the intra-pair correlation coefficient in monozygotic twins, while rDZ indicates the same for dizygotic twins. P-values below 0.05 have been highlighted as significant. Within the analysis, the notation 'A' stands for heritability, 'C' denotes the shared environmental variance component, and 'E' represents the unique environmental variance component. The "model fit" in the table corresponds to the p-value derived from the chi-squared test, which compares the model's log likelihood to the foundational ACE model.

| Variable | rMZ | rDZ | A | C | E | Model fit P-value |
|---|---|---|---|---|---|---|
| **Frontal lobe total thickness** | 0.634 ( 0.417, 0.783 ) | 0.623 ( 0.259, 0.83 ) | 0 | 0.63 (0.454, 0.759) | 0.37 (0.241, 0.546) | 0.736 |
| **Pre-central gyrus total thickness** | 0.727 ( 0.549, 0.841 ) | 0.408 ( -0.026, 0.713 ) | 0 | 0.666 (0.5, 0.784) | 0.334 (0.216, 0.5) | 0.535 |
| **Temporal lobe total thickness** | 0.692 ( 0.499, 0.819 ) | 0.635 ( 0.274, 0.838 ) | 0 | 0.665 (0.501, 0.783) | 0.335 (0.217, 0.499) | 0.375 |
| **Parietal lobe total thickness** | 0.79 ( 0.644, 0.881 ) | 0.392 ( -0.059, 0.708 ) | 0.779 (0.638, 0.865) | 0 | 0.221 (0.135, 0.362) | 0.919 |
| **Post-central gyrus total thickness** | 0.868 ( 0.77, 0.926 ) | 0.632 ( 0.259, 0.839 ) | 0.85 (0.757, 0.908) | 0 | 0.15 (0.092, 0.243) | 0.119 |
| **Occipital lobe total grey matter volume** | 0.895 ( 0.815 0.941 ) | -0.153 ( -0.551 0.303 ) | 0.881 (0.795, 0.93) | 0 | 0.119 (0.07, 0.205) | 1 |

**Table S4: Intra-Pair Correlation Coefficients and Age- and Sex-Adjusted Univariate Analysis of Infratentorial Structure Measurements**

The table merges data from intra-pair correlation coefficients for cortical thickness measurements of key brain regions in monozygotic (MZ) and dizygotic (DZ) twins and the age- and sex-adjusted univariate analysis using volBrain. Results for both segments and their respective 95% confidence intervals (95% CI) are provided. Volume measurements are denoted in millimeters (mm). For clarity, rMZ signifies the intra-pair correlation coefficient in monozygotic twins, while rDZ indicates the same for dizygotic twins. P-values below 0.05 have been highlighted as significant. Within the analysis, the notation 'A' stands for heritability, 'C' denotes the shared environmental variance component, and 'E' represents the unique environmental variance component. The "model fit" in the table corresponds to the p-value derived from the chi-squared test, which compares the model's log likelihood to the foundational ACE model.

The **AE model** was selected for most traits presented in this table, as the analysis revealed very high additive genetic variance (A) and minimal unique environmental variance (E). The “model fit,” also provided in the table, corresponds to the p-value derived from the chi-squared test, comparing the AE model with the foundational ACE model. The results demonstrate that additive genetic factors play a dominant role in infratentorial structures, with little contribution from environmental variance components.

| Variable | rMZ | rDZ | A | C | E | Model fit P-value |
|---|---|---|---|---|---|---|
| **Brain stem volume** | 0.919 ( 0.857 0.955 ) | 0.719 ( 0.418 0.878 ) | 0.926 (0.875, 0.956) | 0 | 0.074 (0.044, 0.125) | 0.154 |
| **Cerebellum total volume** | 0.885 ( 0.797 0.935 ) | 0.71 ( 0.402 0.875 ) | 0.9 (0.833, 0.94) | 0 | 0.1 (0.06, 0.167) | 0.195 |
| **Cerebellar grey matter voluem** | 0.895 ( 0.816 0.942) | 0.709 ( 0.402 0.873) | 0.905 (0.84, 0.942) | 0 | 0.095 (0.058, 0.16) | 0.173 |

The supplementary tables provided here augment the findings discussed in the main manuscript by detailing the statistical underpinnings of the heritability and environmental influences on brain volumes and cortical thickness in twins. These additional insights further support the conclusions drawn in the primary study.
